# **Clinical syndromes and treatment location predict utility of carbapenem sparing therapies in ceftriaxone-non-susceptible*Escherichia coli*bloodstream infection**

**DOI:** 10.1186/s12941-020-00400-z

**Published:** 2020-11-30

**Authors:** Ouli Xie, Kathryn Cisera, Lucy Taylor, Carly Hughes, Benjamin Rogers

**Affiliations:** 1 Department of Microbiology, Monash Health, Clayton, Victoria Australia; 2 Department of Infectious Diseases, Monash Health, Clayton, Victoria Australia; 3 Centre for Inflammatory Diseases, Monash University, Clayton, Victoria Australia

**Keywords:** Bacteremia, Drug resistance, *Escherichia coli* infections, Meropenem, Ceftriaxone

## Abstract

**Background:**

Cefiderocol, ceftazidime-avibactam, ceftolozane-tazobactam, intravenous fosfomycin and plazomicin represent potential carbapenem sparing agents for extended-spectrum-beta-lactamase or AmpC beta-lactamase producing *Escherichia coli* infection. However, available data is limited in predicting the volume of carbapenem therapy which could be substituted and real-world contraindications.

**Methods:**

We determined the number of carbapenem days of therapy (DOT) which could be substituted and frequent contraindications accounting for antimicrobial susceptibility and site of infection in an unselected cohort with ceftriaxone-non-susceptible *E. coli* bacteremia at a single health network from 2015 to 2016. Individual patient data was used to calculate DOT and substitution for each agent.

**Results:**

There were 108 episodes of *E. coli* bacteremia resulting in 67.2 carbapenem DOT/100 patient-days of antimicrobial therapy administered. Ceftazidime-avibactam could be used to substitute 36.2 DOT/100 patient-days (54%) for inpatient definitive therapy, ceftolozane-tazobactam for 34.7 DOT/100 patient-days (52%), cefiderocol for 27.1 DOT/100 patient-days (40%), fosfomycin for 23.3 DOT /100 patient-days (35%) and plazomicin for 27.1 DOT/100 patient-days (40%). Non-urinary tract source of infection was the most frequent contraindication to fosfomycin (25), plazomicin (26) and cefiderocol (26). Use in outpatient parenteral antimicrobial therapy (OPAT) programs accounted for 40% of DOT, all of which could be substituted if stability data allowed for ceftazidime-avibactam and ceftolozane-tazobactam.

**Conclusions:**

All tested agents could be used to replace a significant volume of carbapenem therapy. Establishing stability of these agents for use in OPAT is required for maximizing their use as carbapenem sparing agents while randomized clinical data is awaited for some of these agents in resistant *E. coli* bacteremia.

## Introduction

*Escherichia coli* is one of the leading causes of community onset and nosocomial bloodstream infection [1, 2]. *E. coli* harbouring extended-spectrum beta-lactamases (ESBL) or AmpC beta-lactamases are resistant to oxyiminocephalosporins such as ceftriaxone and have emerged as a global health problem [3]. While Australia has low rates of resistance by global standards, the proportion of *E. coli* harbouring ESBLs have increased between 2013 and 2017 to more than 10% of isolates [2]. In Europe there has been a similar increase in 2018 with more than 15% of isolates resistant to third generation cephalosporins [4].

Carbapenems are the current treatment of choice for ESBL or AmpC producing *E. coli* bloodstream infections. However, rising carbapenem use has been associated with the emergence of carbapenem resistant infections [5]. Amongst other infection control measures, carbapenem sparing strategies offer significant appeal in reducing the emergence of carbapenem resistance.

A randomized clinical trial was unable to demonstrate non-inferiority of piperacillin-tazobactam compared to meropenem for ceftriaxone-resistant *E. coli* or *Klebsiella pneumoniae* bloodstream infection [6]. The new-to-market agents ceftolozane-tazobactam, ceftazidime-avibactam, cefiderocol and plazomicin and the near-market intravenous (IV) fosfomycin each represent other potential carbapenem-sparing antimicrobial options [7–11].

Data from a subset of patients from clinical trials has highlighted ceftolozane-tazobactam, ceftazidime-avibactam, cefiderocol and plazomicin as potentially efficacious agents for ESBL infections [7–10]. There are active clinical trials investigating the efficacy of ceftolozane-tazobactam and IV fosfomycin for resistant *E. coli* bloodstream infection.

To date, studies of these potential carbapenem sparing agents for third-generation cephalosporin non-susceptible infection have analysed data from a subset of patients in clinical trials or ecological studies. Limitations of data from clinical trials include the selected nature of patients and the absence of details of patients who were unable to be treated with the agent. Ecological studies have demonstrated high rates of *in vitro* susceptibility but give little information on the potential clinical application of the agent [11–15]. In practice, some patients may have absolute or relative contraindications to these agents such as allergy, pregnancy, renal impairment, site of infection, or need for delivery of care in an outpatient antimicrobial therapy program (OPAT). Furthermore, selected infections can be treated with alternative agents such as fluoroquinolones which can be delivered orally.

This study aimed to determine the potential for ceftolozane-tazobactam, ceftazidime-avibactam, IV fosfomycin, cefiderocol and plazomicin to be used as an alternative to carbapenem-based therapy for patients with bloodstream infection caused by *E. coli* that are non-susceptible to ceftriaxone by analysis of a real-life patient cohort. Individual patient data was used to determine the volume of carbapenem therapy that could be substituted for each of the potential agents. We also sought to identify frequent patient contraindications or characteristics that may limit the programmatic utility of the agents.

## Material and methods

### Clinical setting

Adult patients (16 years and older) managed by Monash Health for episodes of ceftriaxone non-susceptible *E. coli* bloodstream infection between January 1, 2015 to December 31, 2016 were included in this study. Monash Health is a large Australian tertiary hospital network (1500 adult patient beds) with four acute adult hospitals including two intensive care units. Patients were identified from the laboratory information system. Patients were excluded if their infection was not treated in our health service or their bacterial isolate was not available. For patients with multiple episodes of bloodstream infection, only the first episode during this time-period was included.

### Bacterial isolates

Isolates were identified as *E. coli* by matrix-assisted laser desorption ionization–time of flight mass spectrometry (MALDI-TOF MS, Bruker Daltonik). Susceptibility testing for ceftolozane/tazobactam (bioMerieux), ceftazidime/avibactam (Liofilchem) and plazomicin (Liofilchem) were performed by minimum inhibitory concentration (MIC) strips according to manufacturer instructions [16–18]. Fosfomycin susceptibility testing was performed by disk diffusion (Oxoid) according to EUCAST methods [19]. Cefiderocol susceptibility testing was performed using disks containing 30 µg cefiderocol on standard Mueller-Hinton agar. Breakpoints for these agents were interpreted using EUCAST 2020 clinical breakpoints except for plazomicin MIC (susceptible ≤ 2 µg/mL, intermediate 4 µg/mL, resistant ≥ 8 µg/mL) and cefiderocol disk diffusion zone size (susceptible ≥ 16 mm, intermediate 12–15 mm, resistant ≤ 11 mm) which were interpreted according to FDA interpretive criteria and CLSI 2020 criteria respectively as EUCAST criteria were not available at the time of testing [20–22]. All non-susceptible isolates were re-tested in duplicate.

Susceptibility to other routine antimicrobial agents were ascertained from routine susceptibility testing performed at the time of the bloodstream infection. Our laboratory used VITEK 2 Compact from January to April 2015 and VITEK 2 XL system from May 2015 onwards (bioMerieux) using the VITEK AST-N246 susceptibility card (bioMerieux) for automated susceptibility testing. Ertapenem susceptibility was performed by disk diffusion (Oxoid) according to manufacturer instructions. Results were interpreted using CLSI 2015 and 2016 criteria as these criteria were used for antimicrobial selection at the time this cohort was treated [23, 24].

As a reference method for susceptibility testing (broth microdilution or agar dilution) was not used, we have reported only the category interpretations based on the above standards, rather than MIC values.

### Participants and clinical data

Individual patient data was collected by retrospective chart review. Clinical characteristics included patient demographics, dates of hospitalization, site of infection and all-cause 30-day mortality. Complicated urinary traction infection (UTI) was defined as infection with at least one complicating factor (urinary retention, current indwelling catheter, obstructive uropathy, or any functional or anatomical abnormality including ureteric stents).

Duration of antimicrobial agents used for empiric and definitive therapy were recorded including any treatment on our health service OPAT program. Empiric and definitive therapy were defined as antimicrobial therapy before and after availability of formal antimicrobial susceptibility results, respectively. Our health-service does not use piperacillin/tazobactam for therapy of ceftriaxone-non-susceptible *E. coli*. Susceptibility results are suppressed by the laboratory. A subset of patients during the study period were participants in the MERINO trial [6]. For participants who were randomized to piperacillin-tazobactam, they were considered to have received standard of care carbapenem therapy for the purposes of this analysis.

Contraindications to the agents of interest and polymicrobial bloodstream infection requiring addition of another agent was also recorded. Medical contraindications were not counted for cases where the isolate was resistant to the agent of interest. Allergy history was obtained from pharmacist completed assessments on admission and from the medical admission notes.

A rash or severe reaction (severe cutaneous adverse reaction, anaphylaxis or angioedema) to the antimicrobial of interest was considered a contraindication. History of a rash to any cephalosporin or a severe reaction to any beta-lactam was recorded as a contraindication to ceftolozane-tazobactam, ceftazidime-avibactam and cefiderocol. Allergy to piperacillin-tazobactam was considered a contraindication to ceftolozane-tazobactam.

Other indications and contraindications were derived from clinical trials utilising the agents of interest. Pregnancy was considered a contraindication to ceftazidime-avibactam, plazomicin and cefiderocol due to a lack of safety data. Renal dysfunction at the time of bloodstream infection defined as an eGFR < 30 mL/min/1.73m^2^ calculated by CKD-EPI was a contraindication to plazomicin [9]. Cirrhosis, renal impairment requiring dialysis, acute decompensated heart failure or NYHA class IV heart failure was considered a contraindication to IV fosfomycin due to the sodium load associated with IV fosfomycin [25]. Infections not from the urinary tract were also considered contraindications for plazomicin, cefiderocol and fosfomycin due to lack of clinical data.

Suitability for OPAT was based on the ability to deliver the drug with a single daily treatment (intermittent infusion, or continuous infusion stable for 24 hours at room temperature). Based on this definition, the only drug definitively suitable for OPAT was plazomicin.

### Analysis

The primary outcome was the number of days of carbapenem exposure potentially avoided per 100 patient-days of antimicrobial therapy (pd), with each of the proposed agents when used for definitive therapy. The days of therapy (DOT) which could be substituted with each agent was the number of carbapenem days administered for definitive therapy, less the days where isolates were not susceptible to the agent or where there were contraindications for use of the agent including site of infection. DOT for carbapenems was calculated by retrospective chart review according to the number of days where at least one dose of carbapenem was administered. Total patient-days of antimicrobial therapy was determined by the number of days when any antibiotic was administered for the episode of bacteremia of interest.

Secondary outcomes were: (i) frequencies of contraindications to therapy with the proposed agents, (ii) increase in carbapenem DOT spared if these agents were stable for administration in an OPAT setting, (iii) increase in carbapenem DOT spared if these agents were substituted for empiric therapy, and (iv) the need for additional antimicrobial agents for cases of polymicrobial infection.

## Results

**Fig. 1:**
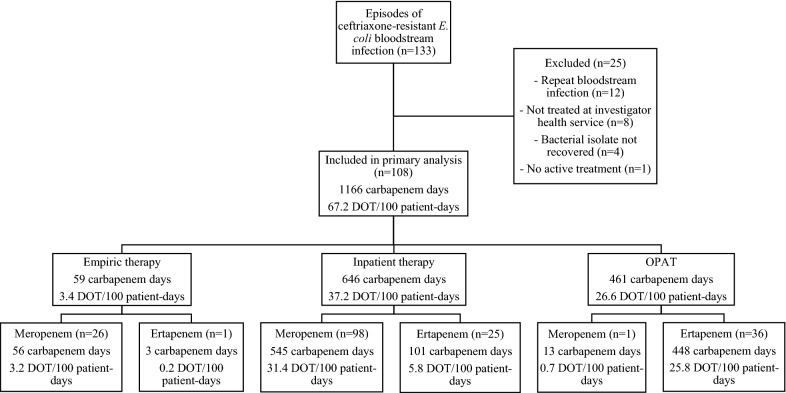
Details of Carbapenem Therapy.*DOT* days of therapy,* OPAT* outpatient parenteral antimicrobial therapy

There were 108 unique episodes of ceftriaxone-non-susceptible *E. coli* bloodstream infection between Jan 1, 2015 and Dec 31, 2016 (Fig. 1). Twenty-five cases were excluded, of which 12 cases were repeat episodes of bloodstream infection, eight cases were not treated in our health-service, four bacterial isolates were non-recoverable, and one case did not receive active treatment. The most common source was UTI, of which 46 were complicated UTI and 35 uncomplicated UTI. The median duration of treatment was 15 days (IQR 12, 17) for a total of 1736 DOT. Follow-up at 30 days was available for 99 (92%) patients. All-cause mortality at 30 days was 11% (11/99). Demographics and characteristics of cases are described in Table 1.

**Table 1:** Demographics of patients and characteristics of bloodstream infection

Characteristic	
Male	52 (48%)
Median age, years	71 (IQR 57, 83)
Median days in hospital before bloodstream infection	0 (IQR 0, 1)
Median inpatient days after bloodstream infection	8 (IQR 5, 12)
Number admitted to OPAT	38 (35%)
Polymicrobial bloodstream infection^a^	6 (6%)
Source of infection	
Complicated UTI	46 (43%)
Uncomplicated UTI	35 (32%)
Intra-abdominal other than biliary tract	11 (10%)
Biliary tract	7 (6%)
Febrile neutropenia	4 (4%)
No focus	4 (4%)
Native osteomyelitis	1 (1%)
Total duration of antimicrobial therapy, median, days	15 (IQR 12, 17)
Number treated with carbapenem	102 (94%)
Number de-escalated to oral therapy^b^	45 (42%)
Median duration oral therapy, days	12 (IQR 6, 15)
30-day mortality^c^	11/99 (11%)

*OPAT* outpatient antimicrobial therapy,* UTI* urinary tract infection

^a^ 1 patient was bacteremic with both *S. agalactiae* and *P. mirabilis*, 2 patients had *Staphylococcus aureus*, 1 patient had *Streptococcus anginosus group*, 1 patient had *Pseudomonas aeruginosa*, 1 patient had *Enterococcus raffinosus*

^b^ 29 patients given ciprofloxacin, 10 trimethoprim-sulfamethoxazole, 3 trimethoprim, 2 oral fosfomycin, 1 nitrofurantoin,

^c^ 30-day follow-up not available for 9 patients

### Antimicrobial therapy Used

A total of 1107 carbapenem days (63.8 DOT/100 pd) were used for definitive therapy. Meropenem was the most used carbapenem and was used in 98 (91%) patients for definitive therapy accounting for a total of 545 antibiotic days or 31.4 DOT/100 pd (Fig. 1). This included six patients who received piperacillin-tazobactam as part of the MERINO trial. For the purpose of further analysis, these patients were considered to have received inpatient meropenem or ertapenem for OPAT (if susceptible) as the standard of care. Empiric carbapenem therapy was used for 27 (25%) patients accounting for 59 carbapenem days (3.4 DOT/100 pd). A total of 1166 carbapenem days (67.2 DOT/100 pd) were used including empiric and definitive therapy. OPAT was used in 37 cases (34%) comprising 461 carbapenem days (26.6 DOT/100 pd). Oral antimicrobials were used to complete therapy in 45 (42%) patients. Twenty-nine (27%) patients were treated with oral ciprofloxacin, 10 (9%) patients to trimethoprim-sulfamethoxazole and six (6%) patients to other therapies. Six (6%) patients did not receive any carbapenem therapy.

### Antimicrobial susceptibility

All isolates were susceptible to ceftazidime-avibactam, plazomicin and cefiderocol. Three isolates (3%) were resistant to ceftolozane-tazobactam and four isolates (4%) were resistant to fosfomycin (Table 2). Only 43 (40%) isolates were susceptible to ciprofloxacin and 44 (41%) isolates to trimethoprim-sulfamethoxazole.

**Table 2:** Susceptibilities

Antimicrobial	Number susceptible (n = 108)
*New agents*	
Cefiderocol	108 (100%)
Ceftazidime-avibactam	108 (100%)
Ceftolozane-tazobactam	105 (97%)
Fosfomycin	104 (96%)
Plazomicin	108 (100%)
*Standard agents*	
Amikacin	108 (100%)
Gentamicin	72 (67%)
Tobramycin	61 (56%)
Piperacillin-tazobactam	99 (92%)
Ceftazidime	46 (43%)
Ceftriaxone	0 (0%)
Ertapenem	103 (95%)
Meropenem	108 (100%)
Ciprofloxacin	43 (40%)
Trimethoprim-sulfamethoxazole	44 (41%)

### Indications and contraindications for carbapenem sparing agents

Ceftolozane-tazobactam, ceftazidime-avibactam and cefiderocol were contraindicated in due to a cephalosporin rash for one patient and penicillin anaphylaxis/angioedema in another patient (Table 3). The most common contraindication was non-urinary tract source in 25 (23%) patients for fosfomycin, 26 (24%) for plazomicin and 26 (24%) for cefiderocol. Plazomicin was contraindicated in 21 (19%) patients with an eGFR < 30 mL/min/1.73 m^2^. Fosfomycin was contraindicated in four (4%) patients due to decompensated or NYHA class IV heart failure and three (3%) patients due to cirrhosis. There were no patients who were pregnant in the cohort.

**Table 3:** Contraindications to carbapenem sparing agents

Antimicrobial	Total	Rash	Anaphylaxis	SCAR	Pregnancy	Heart failure^a^	Cirrhosis	Non-urinary tract source	Renal impairment^e^
Ceftolozane-tazobactam	2	1	1	0	–	–	–	–	–
Ceftazidime-avibactam	2	1	1	0	0	–	–	–	–
Plazomicin^b^	42	0	0	0	0	–	–	26	21
Fosfomycin^c^	32	0	0	0	–	4	3	25	1
Cefiderocol^d^	26	1	1	0	0	–	–	26	–

*SCAR* severe cutaneous adverse reaction

Excludes cases which tested resistant *in vitro*

^a^ Includes both acute decompensated heart failure and NYHA IV heart failure

^b^ 5 patients had both non-urinary tract source and renal impairment (eGFR < 30 mL/min/1.73 m^2^) as contraindications

^c^ 1 patient had both cirrhosis and was dialysis dependent

^d^ 2 patients had both allergy and non-urinary tract source

^e^ Dialysis dependence for fosfomycin and eGFR < 30 mL/min/1.73 m^2^ for plazomicin

Polymicrobial bloodstream infection was present in six (6%) patients. In patients where carbapenem substitution was possible, additional antimicrobial therapy would have been required for two patients if treated with ceftazidime-avibactam (*Enterococcus raffinosus* 17 antibiotic days, *Staphylococcus aureus* 41 antibiotic days), one patient for cefiderocol (*S. aureus* 41 antibiotic days), one patient for ceftolozane/tazobactam (*S. aureus* 41 antibiotic days), and one patient for fosfomycin (*S. aureus* 41 antibiotic days).

### Carbapenem sparing therapy

**Fig. 2:**
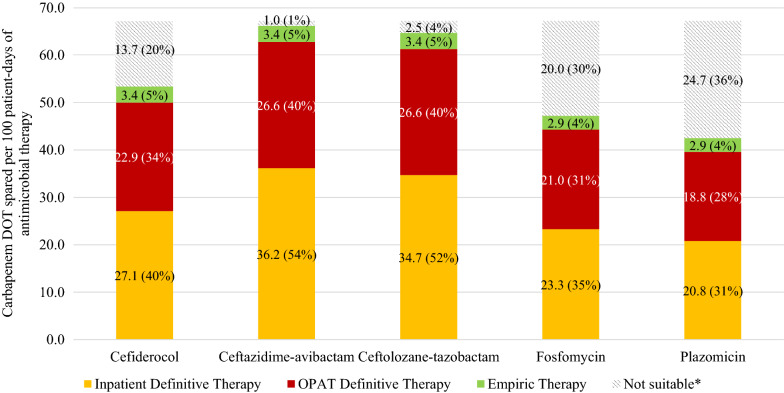
Potential carbapenem days spared.  *DOT* days of therapy. *Due to resistance, site of infection or contraindications. Individual bars represent the DOT per 100 patient-days of antimicrobial therapy and percentage of potential carbapenem days spared for each agent divided into setting of administration and empiric vs definitive therapy

Ceftazidime-avibactam could be used as a carbapenem sparing agent most frequently in 100 patients (93% of all patients). Inpatient definitive therapy could spare 36.2 carbapenem DOT/100 pd (629 days, 54% of all carbapenem days, median 5 days/patient) (Fig. 2). Ceftolozane-tazobactam could be used in 96 patients (89%) after excluding resistant isolates and contraindications. Inpatient definitive therapy could spare 34.7 DOT/100 pd (602 carbapenem days, 52%, median 5 days/patient). Cefiderocol could be used for 76 patients (70%) and could spare 27.1 DOT/100 pd (471 carbapenem days, 40%, median 4 days/patient). Fosfomycin could be used in 66 patients (61%) and could spare 23.3 DOT/100 pd (404 carbapenem days, 35%, median 3 days/patient). Plazomicin could be used in 60 patients (56%), including OPAT where it is stable for once daily administration. Inpatient definitive therapy could spare 20.8 DOT/100 pd (361 carbapenem days, 31%, median 2 days/patient). Including OPAT, plazomicin could spare 39.6 DOT/100 pd (688 carbapenem days, 58%, median 3 days/patient) for definitive therapy.

With the addition of empiric therapy, ceftazidime-avibactam, ceftolozane-tazobactam and cefiderocol could be used to spare an additional 3.4 DOT/100 pd (59 carbapenem days, 5%), fosfomycin could spare 2.9 DOT/100 patient-days (51 days, 4%) and plazomicin could spare 2.9 DOT/100 pd (50 days, 4%).

### Additional carbapenem sparing therapy if stable for administration in OPAT

Stability data currently only supports the use of plazomicin in OPAT. If stable to administer in an OPAT setting, ceftazidime-avibactam could be used to spare an additional 26.6 DOT/100 pd (461 carbapenem days, 40%) totalling 62.8 DOT/100 pd (1090 days, 94%, median 9 days/patient) for definitive therapy. Ceftolozane-tazobactam could also spare an additional 26.6 DOT/100 pd (461 days, 40%) totalling 61.3 DOT/100 pd (1064 days, 91%, median 8 days/patient) for definitive therapy. Cefiderocol could spare an additional 22.9 DOT/100 pd (398 days, 34%) totalling 50.1 DOT/100 pd (869 days, 75%, median 5 days/patient) and fosfomycin could spare an additional 21.0 DOT/100 pd (364 days, 31%) totalling 44.2 DOT/100 pd (768 days, 66%, median 4 days/patient).

## Discussion

In the setting of increasing carbapenem use and resistance, carbapenem sparing agents are an appealing option for the treatment of ESBL or AmpC producing *E. coli*. In an unselected cohort of 108 episodes of ceftriaxone-non-susceptible *E. coli* bloodstream infection, patients were exposed to 1166 total days of carbapenem therapy or 67.2 carbapenem DOT/100 pd for the treatment of these infections. *In vitro* susceptibility to ceftazidime-avibactam, ceftolozane-tazobactam, IV fosfomycin, plazomicin and cefiderocol were high and similar to that reported for global isolates of ESBL producing Enterobacterales [11–15]. Ceftazidime-avibactam had the potential to replace the greatest number of carbapenem days for inpatient definitive therapy (36.2 DOT/100 pd, 54% of all carbapenem days) while plazomicin had the lowest at 20.8 DOT/100 pd (31%). However, accounting for suitability for administration in an OPAT setting, plazomicin could be used to spare 39.6 carbapenem DOT/100 pd (58%) while stability data is lacking for other agents limiting their use. To the best of our knowledge, this is the only study comparing potential utility of these agents across an unselected cohort of patients with ceftriaxone-non-susceptible *E. coli* bloodstream infection.

Drug stability for prolonged infusion in the OPAT setting was an important determinant of potential carbapenem replacement with 26.6 carbapenem DOT/100 pd (461 carbapenem days) administered in OPAT of which 100% could be substituted with ceftazidime-avibactam or ceftolozane-tazobactam if stability data allowed. Ceftazidime-avibactam is reported to be stable for 12 hours at room temperature and may be able to be administered using two infusions over 24 hours [26]. However, experience with ceftazidime-avibactam in OPAT is limited and further stability studies are required to expand its use. Cefiderocol is only stable for four hours at room temperature and is not currently stable for use in OPAT [27]. A small case series has used infusions of ceftolozane-tazobactam over 24 hours in OPAT with good outcomes [28]. However, stability studies have shown ceftolozane-tazobactam to be stable for only 12 hours at 32 °C [29]. Allowing for this, administration with two infusions over 24 hours in OPAT is still feasible. Little information is available regarding stability of IV fosfomycin for use in OPAT. A completed clinical trial and an ongoing trial of IV fosfomycin used infusions every six-eight hours administered over one hour [25, 30]. Plazomicin is administered daily and is compatible with OPAT.

Use of IV fosfomycin, cefiderocol and plazomicin is limited by the source of infection. IV fosfomycin has been found to be effective for complicated urinary tract infection or pyelonephritis [30]. The trial included 19 patients with bloodstream infection treated with IV fosfomycin. The results of the FOREST trial are awaited for efficacy of IV fosfomycin in patients with multidrug resistant *E. coli* bloodstream infection from a urinary tract source. Whilst fosfomycin demonstrates high tissue penetration, use in infection with sources outside of the urinary tract are limited to observational studies and often in combination with other agents [31]. Of the 32 patients who could not use fosfomycin, 25 had a non-urinary tract source.

Similarly, plazomicin and cefiderocol have been found to be effective for complicated urinary tract infections but robust evidence is lacking for infection from other sources [9, 10]. A smaller trial of plazomicin in carbapenem-resistant infections included predominantly patients assessed to have primary bacteremia [32]. Twenty-six of the 42 patients who could not use plazomicin had a non-urinary tract source and 21 patients had an eGFR < 30 mL/min/1.73 m. Plazomicin may have lower rates of nephrotoxicity compared to previous generation aminoglycosides, but real world experience in patients with advanced kidney disease is lacking [33]. All patients who could not use cefiderocol had a non-urinary tract source. Further trials and results for cefiderocol are forthcoming for carbapenem resistant infection and nosocomial pneumonia.

Rates of penicillin anaphylaxis/angioedema as contraindications to cefiderocol, ceftazidime-avibactam and ceftolozane-tazobactam were low and comparable to previous literature on beta-lactam allergy in the general community [34].

It is also important to note, that while these agents could be used as carbapenem sparing agents for ceftriaxone-resistant *E. coli* isolated in blood culture, additional antimicrobials may be required for polymicrobial infection. Intra-abdominal and biliary sources of infection were found for 18 patients and six patients had polymicrobial bloodstream infection. All five agents investigated as carbapenem sparing agents in this study lack anerobic activity and agents such as metronidazole may need to be added for adequate cover. Cefiderocol has limited gram positive activity and if considered for use for intra-abdominal infection would require additional cover for streptococci [35]. Four patients had febrile neutropenia and additional cover for *Staphylococcus aureus* may be warranted if using these agents at least for empiric therapy [36].

While a substantial proportion of carbapenem therapy could be spared with the use of these novel agents, stewardship is also required. Ceftazidime-avibactam and cefiderocol are active against some carbapenemase producing Gram negatives and should be reserved for infections with few other options [37, 38]. Plazomicin has also been used for carbapenem-resistant infection and should similarly be reserved for this indication [32]. While IV fosfomycin has been used in the treatment for carbapenemase producing Gram negative infection, it is usually used in combination with another agent [39]. Ceftolozane-tazobactam has enhanced activity against *Pseudomonas* but is not active against carbapenemase producing Gram negatives [40]. Ceftolozane-tazobactam and IV fosfomycin therefore are attractive options as carbapenem sparing agents. Given a small proportion of isolates are resistant, use as definitive therapy after availability of susceptibility results is most appropriate.

Cost and availability must also be considered for the potential use of these carbapenem sparing agents. At the time of writing, plazomicin and cefiderocol were not available in our region. Where these agents may not be available, methodology of this study could be applied to other approaches such as early transition to susceptible oral agents or use of aminoglycosides for urinary tract source based on local epidemiology.

A limitation of this study is the use of a non-reference method for susceptibility testing of isolates. MIC results for the agents tested have therefore not been reported. However, testing by MIC strips and disk diffusion reflects real-world practice in our clinical laboratory (and most others) and the basis on which these agents would be used in clinical care. Ascertainment of indications and contraindications were also performed retrospectively.

Additionally, the number of carbapenem days spared is based on antimicrobial prescribing practices in 2015–2016. Recent studies have suggested non-inferiority of shorter durations of treatment for Gram-negative bloodstream infections, particularly from urinary tract sources [41]. Therefore, the number of carbapenem days spared may be less than what is estimated in our study.

Adverse events while on therapy may also occur for these novel agents and could reduce their real-world compared to the estimates in this study. We expect the beta-lactam antibiotics to be well tolerated but toxicity may occur with prolonged aminoglycoside use such as with plazomicin.

## Conclusion

In summary, we found the agents cefiderocol, ceftazidime-avibactam, ceftolozane-tazobactam, IV fosfomycin and plazomicin could be used to spare between 31–58% of carbapenem use for ceftriaxone-non-susceptible *E. coli* bloodstream infection. Carbapenem use in OPAT was significant and further studies on the stability of these agents for OPAT is required. Further studies examining the use of IV fosfomycin, cefiderocol and plazomicin in infections other than from a urinary tract source could also increase their utility as carbapenem sparing agents while randomized evidence is awaited for the use of IV fosfomycin and IV ceftolozane-tazobactam in resistant *E. coli* bloodstream infection.

## Data Availability

The datasets generated and/or analysed during the current study are available from the corresponding author on reasonable request.

## Abbreviations

ESBL: Extended-spectrum beta-lactamase
IV: Intravenous
MIC: Minimum inhibitory concentration
OPAT: Outpatient antimicrobial therapy program
UTI: Urinary tract infection
pd: Patient-days of antimicrobial therapy
DOT: Days of therapy
